# Triglyceride-glucose index and short-term mortality of adult patients with sepsis: a meta-analysis

**DOI:** 10.3389/fendo.2026.1875452

**Published:** 2026-07-30

**Authors:** Weijie Wang, Wei Zheng, Yuanyuan Cai, Fenfen Shen

**Affiliations:** Department of ICU, First People’s Hospital of Daishan County, Zhoushan, China

**Keywords:** meta-analysis, mortality, prognosis, sepsis, triglyceride-glucose index

## Abstract

**Background:**

The triglyceride–glucose (TyG) index, a surrogate marker of insulin resistance, has been associated with outcomes in sepsis, but existing evidence is inconsistent. This meta-analysis aimed to evaluate the association between a high TyG index and short-term mortality in adult patients with sepsis.

**Methods:**

A systematic search of PubMed, Embase, Web of Science, Wanfang, and CNKI was conducted to identify observational studies reporting the association between TyG index and short-term mortality (in-hospital or ≤30-day) in adult sepsis patients. Effect estimates were pooled using a random-effects model accounting for the influence of heterogeneity.

**Results:**

Eight retrospective cohort studies involving 14,772 patients were included. Overall, a higher TyG index was associated with increased short-term mortality (odds ratio [OR] 1.72, 95% confidence intervals 1.44–2.04; I² = 53%). Subgroup analyses showed consistent associations across mean age, proportion of men, and diabetic status (non-diabetic vs. mixed populations: *p* for subgroup difference all > 0.05). The association remained significant regardless of cutoff determination methods, cutoff values, outcome type (in-hospital vs. 28-day mortality), and study quality scores (*p* for subgroup difference all > 0.05). Meta-regression analyses did not suggest the association was significantly modified by mean age, proportion of men, proportion of diabetic patients, cutoff values of TyG index, or study quality scores (*p* all > 0.05).

**Conclusions:**

A higher TyG index is associated with an increased risk of short-term mortality in adult patients with sepsis, supporting its potential role as a simple prognostic marker in clinical practice.

**Systematic Review Registration:**

https://www.crd.york.ac.uk/prospero/, identifier CRD420261389660.

## Introduction

Sepsis is a life-threatening syndrome characterized by a dysregulated host response to infection leading to organ dysfunction, and it remains a major global health burden (1). Recent epidemiological data indicate that sepsis affects tens of millions of people worldwide each year and is associated with substantial morbidity and mortality, particularly in critically ill populations (2, 3). Despite advances in supportive care and early recognition strategies, short-term mortality in sepsis remains unacceptably high (4, 5). Early risk stratification is therefore essential for guiding clinical decision-making and optimizing resource allocation (4, 5). Although established scoring systems such as Sequential Organ Failure Assessment (SOFA) and Acute Physiology and Chronic Health Evaluation II (APACHE II) are widely used, they are relatively complex and may not fully capture the metabolic alterations that are increasingly recognized as key contributors to sepsis pathophysiology (6, 7). Accordingly, identifying simple, reliable, and readily available biomarkers for predicting short-term mortality in sepsis remains an important unmet clinical need.

The triglyceride–glucose (TyG) index, calculated as Ln [fasting triglycerides (mg/dL) × fasting glucose (mg/dL)/2], has emerged as a practical surrogate marker of insulin resistance and metabolic dysfunction (8). It is inexpensive, easily obtainable from routine laboratory tests, and has demonstrated prognostic value across various cardiovascular and critical illness settings (9–11). Mechanistically, an elevated TyG index likely reflects integrated metabolic and inflammatory dysregulation, including insulin resistance, endothelial dysfunction, and oxidative stress (12, 13), all of which may exacerbate organ injury and contribute to poor outcomes in sepsis. In recent years, several observational studies have explored the association between TyG index and mortality in sepsis (14–21). However, the findings have been inconsistent, likely due to differences in study design, population characteristics, and cutoff definitions. To date, no comprehensive synthesis of the available evidence has been performed. Therefore, the present meta-analysis aimed to systematically evaluate the association between a high TyG index and short-term mortality in adult patients with sepsis, and to explore the potential influence of study characteristics on the association.

## Methods

The meta-analysis was conducted in accordance with established methodological guidance, following the principles outlined in the PRISMA 2020 statement (22) and the Cochrane Handbook for Systematic Reviews of Interventions (22), encompassing protocol planning, study selection, data collection, statistical analysis, and results interpretation. The study protocol was registered prospectively in the PROSPERO database (registration number: CRD420261389660).

### Database search

A systematic literature search was conducted in PubMed, Embase, Web of Science, Wanfang, and China National Knowledge Infrastructure (CNKI) to identify studies that met the eligibility criteria for inclusion. The search strategy was constructed using the combination of the following terms (1): “TyG index” OR “triglyceride-glucose index” OR “triglyceride and glucose index” OR “triglyceride glucose index” OR “triacylglycerol glucose index”; and (2) “sepsis” OR “septic” OR “septicemia”. Only full-text articles published in English or Chinese and involving human participants were eligible for inclusion. Additionally, the reference lists of relevant reviews and original studies were manually examined to identify further potentially eligible publications. All databases were searched from their inception up to March 28, 2026. Detailed search strategies for each database are presented in **Supplementary File 1**.

### Study inclusion and exclusion criteria

The selection of studies was guided by the PICOS principle:

P (Population): Adult patients (≥ 18 years) diagnosed with sepsis according to recognized criteria.

I (Exposure): TyG index assessed as a categorical variable (e.g., high vs. low, or according to tertiles, quartiles, or study-defined cutoffs), measured at baseline or early during admission. The cutoff for defining a high TyG index was consistent with that used in the original studies. Patients with higher TyG index were considered the exposed group.

C (Comparator): Patients in the low TyG index category (or corresponding reference group) compared with those in higher TyG index categories.

Outcome (O): Short-term mortality, defined as all-cause mortality within 30 days after admission, which typically included 28-day, 30-day, or in-hospital mortality.

S (Study design): Observational studies with longitudinal follow-up, including prospective or retrospective cohort studies, nested case-control studies, or *post-hoc* analyses of clinical trials.

Exclusion criteria include studies involving pediatric populations, non-sepsis or mixed populations without extractable sepsis-specific data, studies lacking TyG index or mortality outcomes, or insufficient data to estimate effect sizes; as well as reviews, editorials, letters, case reports, and animal or *in vitro* studies. When multiple studies involve overlapping populations, only the study with the largest sample size was included.

### Study quality assessment

Two reviewers independently performed the literature search, screened studies for eligibility, extracted data, and assessed study quality. Any disagreements were resolved through discussion, and when necessary, a third investigator was consulted to reach consensus. The methodological quality of the included studies was evaluated using the Newcastle–Ottawa Scale (NOS) (23), which assesses study quality across three domains: selection, comparability, and outcome assessment. NOS scores range from 1 to 9, with studies scoring ≥ 8 considered to be of high quality.

### Data collection

Data extraction was conducted independently by two reviewers using a standardized and pre-tested data collection form. Extracted variables included study characteristics (first author, publication year, country, and study design), participant characteristics (diagnostic criteria for sepsis, mean age, sex distribution, and diabetic status), exposure details (timing of TyG index measurement, methods for determining the cutoffs of TyG index, and cutoff values for defining high TyG index), follow-up durations, number of patients who died during follow-up, and variables adjusted for in the analyses examining the association between TyG index and short-term mortality of patients with sepsis.

### Statistical analyses

The association between TyG index and short-term mortality of patients with sepsis was evaluated by pooling odds ratios (ORs) with their corresponding 95% confidence intervals (CIs) (22), compared between patients with a high vs. low level of TyG index. If data from multiple models were available, those from the most adequately adjusted model were extracted for subsequent analysis. When necessary, effect estimates and their standard errors were derived from the reported 95% CIs or *p* values. Before pooling, all effect measures were converted to the natural logarithmic scale to improve normality and ensure variance stabilization for meta-analysis (22). To evaluate variability across studies, we applied the Cochrane Q test and calculated the I² statistic (24). Heterogeneity was categorized based on I² values as low (< 25%), moderate (25–75%), or high (> 75%). Pooled effect estimates were calculated using the inverse variance (IV) approach within a random-effects framework (DerSimonian–Laird method) to account for potential between-study variability (22). Sensitivity analyses were performed using a leave-one-out approach, in which each study was sequentially excluded to assess the stability and robustness of the pooled results (25). To identify possible sources of between-study variability, we performed predefined subgroup analyses stratified by mean ages of the patients, proportions of men, diabetic status, methods for determining the cutoffs of TyG index, cutoff values of TyG index, outcomes reported (in-hospital vs. 28-day mortality), and study quality scores. Medians of continuous variables were chosen to define subgroups in order to balance the number of studies in each subgroup. Additionally, univariate meta-regression analyses were conducted to examine the potential impact of study-level characteristics on the association between TyG index and short-term mortality of patients with sepsis, such as mean age, proportion of men, proportion of patients with diabetes, cutoff value of TyG index, and NOS scores (22). Publication bias was assessed through visual inspection of funnel plot symmetry and further evaluated using Egger’s regression test (26). A two-sided *p* value < 0.05 was considered indicative of statistical significance. All analyses were performed using RevMan (version 5.3; Cochrane Collaboration, Oxford, UK) and Stata (version 17.0; StataCorp, College Station, TX, USA).

## Results

### Database search results

**Figure 1:**
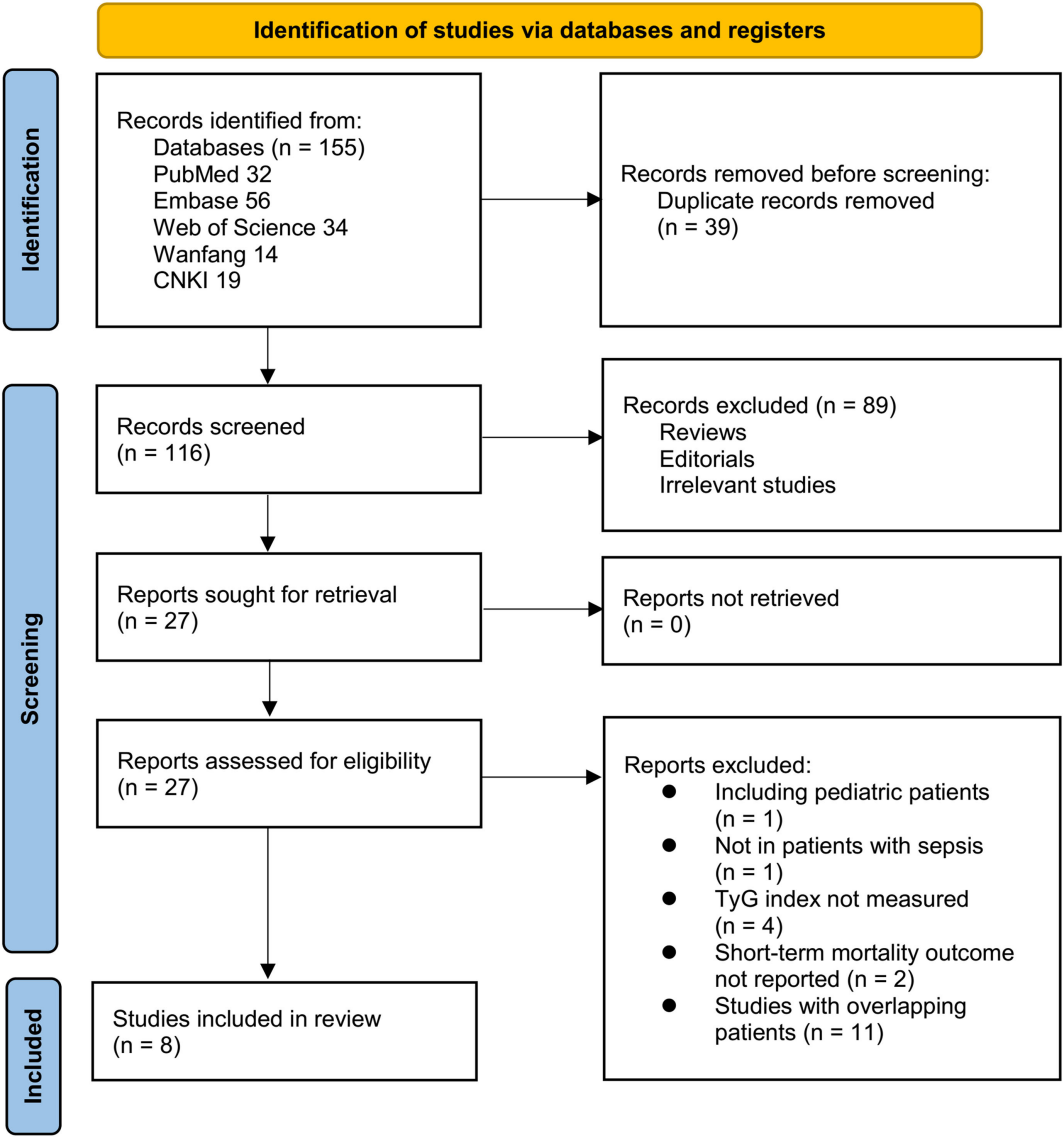
Flow diagram of the study selection process.

The study selection process is illustrated in **Figure 1**. A total of 155 records were retrieved from the 5 databases, of which 39 duplicates were removed. Following title and abstract screening, 89 records were excluded for not meeting the predefined inclusion criteria. The full texts of 27 articles were subsequently evaluated independently by 2 reviewers, and 19 were excluded for the reasons detailed in **Figure 1**. Notably, 11 studies were excluded because they were based on overlapping populations derived from the MIMIC-IV database, and only the largest dataset was retained to avoid duplication of participants. Details of these excluded studies are provided in **Supplementary File 2**. Ultimately, 8 studies met the eligibility criteria and were included in the quantitative meta-analysis (14–21).

### Overview of study characteristics

The main characteristics of the included studies are summarized in **Table 1**. A total of 8 retrospective cohort studies (14–21) published between 2022 and 2026 were included, comprising 14,772 adult patients with sepsis. All studies enrolled adult patients diagnosed with sepsis according to Sepsis-3.0 criteria. The included studies were conducted across multiple geographic regions, including China (15, 16, 19–21), the United States (17), and Turkey (14, 18). The sample sizes ranged from 98 to 8,840 patients, indicating substantial variability in study scale. The mean age of participants ranged from 43.8 to 78.0 years, and the proportion of men ranged from 52.0% to 84.0%, suggesting a predominantly middle-aged to elderly and male population. The proportion of patients with diabetes varied considerably across studies (0% to 64.9%). The TyG index was consistently measured at baseline, most commonly within 24 hours of admission under fasting conditions (14, 15, 17, 18, 20, 21), although two studies used the first fasting blood sampling after admission (16, 19). Various methods were used to determine TyG cutoff values, including receiver operating characteristic (ROC) curve analysis (14, 15, 18–20), quartile comparisons (Q4 vs. Q1) (17), and tertile comparisons (T3 vs. T1) (16, 21) with cutoff values generally ranging from 8.54 to 9.55. Follow-up duration focused on short-term outcomes, including in-hospital mortality (15–17, 21) and 28-day mortality (14, 18–20). Accordingly, a total of 2,932 (19.8%) patients died during follow-up. All studies reported multivariable-adjusted estimates, controlling for key confounders such as age, sex, comorbidities, disease severity scores (e.g., SOFA, APACHE II), and laboratory parameters, although the extent of adjustment varied across studies.

**Table 1:** Characteristics of the included studies.

Study	Country	Study design	Sample size	Diagnostic criteria for sepsis	Mean age (years)	Men (%)	DM (%)	Timing of TyG index evaluation	Methods for determining cutoffs of TyG index	Cutoff value of TyG index	Follow-up duration (days)	No. of patients died	Variables adjusted
Liu 2022 (15)	China	RC	98	Sepsis-3.0 criteria	67.5	63.3	0	Within 24 h of admission (fasting status)	ROC curve analysis	9.48	In-hospital	16	Age, sex, HR, MAP, PCT, CRP, LAC, IL-6, MV, SOFA, and APACHE II scores
Lv 2024 (17)	USA	RC	8840	Sepsis-3.0 criteria	66.6	58.6	32.8	Within 24 h of admission (fasting status)	Q4:Q1	9.55	In-hospital	1251	Age, sex, current smoking, BMI, abdominal obesity, comorbidities, and SOFA score
Elmas 2025 (14)	Turkey	RC	208	Sepsis-3.0 criteria	78.0	52.0	64.9	Within 24 h of admission (fasting status)	ROC curve analysis	9.10	28	89	Age, sex, ALB, HbA1c, HDL-C, DM status, SOFA, and APACHE II scores
Sahin 2026 (18)	Turkey	RC	600	Sepsis-3.0 criteria	65.8	60.0	34.3	Within 24 h of admission (fasting status)	ROC curve analysis	8.95	28	240	Age, sex, ALB, LAC, PCT, septic shock, comorbidities, and SOFA score
Wu 2026 (19)	China	RC	1278	Sepsis-3.0 criteria	43.8	84.0	6.4	First fasting blood sampling after admission	ROC curve analysis	9.22	28	231	Age, sex, comorbidities, and SOFA score
Liu 2026 (16)	China	RC	200	Sepsis-3.0 criteria	68.1	79.0	34	First fasting blood sampling after admission	T3:T1	9.45	In-hospital	122	Age, sex, CAD, and maximal PCT
Zhang 2026a (20)	China	RC	578	Sepsis-3.0 criteria	59.0	65.6	0	Within 24 h of admission (fasting status)	ROC curve analysis	8.54	28	246	Age, sex, BMI, comorbidities, HR, MAP, HBG, CRP, ALB, LAC, SOFA, and APACHE II scores
Zhang 2026b (21)	China	RC	2970	Sepsis-3.0 criteria	68.0	62.5	35.3	Within 24 h of admission (fasting status)	T3:T1	9.31	In-hospital	737	Age, sex, hypertension, sepsis shock, DM, PLT, WBC, TBIL, PCT, and HCT

### Study quality evaluation

The methodological quality of the included studies was assessed using the NOS, with detailed results presented in **Table 2**. The NOS scores ranged from 7 to 9, indicating moderate to high overall study quality. Two studies (14, 18) achieved the maximum score of 9, reflecting strong methodological rigor, including appropriate cohort selection, adequate control for confounders, and reliable exposure and outcome assessment. The majority of studies (n=5) scored 8 (16, 17, 19–21), suggesting generally robust quality with minor limitations, such as incomplete representativeness of the exposed cohort or insufficient follow-up duration. One study (15) received a score of 7, mainly due to limited representativeness and shorter follow-up. Overall, exposure ascertainment and outcome assessment were appropriate across studies, and most analyses adjusted for age and multiple confounding variables. Taken together, the included studies were considered to be of moderate to high quality, supporting the reliability of the pooled results while acknowledging some heterogeneity in study design and adjustment strategies.

**Table 2:** Study quality evaluation via the Newcastle-Ottawa scale.

Study	Representativeness of the exposed cohort	Selection of the non-exposed cohort	Ascertainment of exposure	Outcome not present at baseline	Control for age	Control for other confounding factors	Assessment of outcome	Enough long follow-up duration	Adequacy of follow-up of cohorts	Total
Liu 2022 (15)	0	1	1	1	1	1	1	0	1	7
Lv 2024 (17)	1	1	1	1	1	1	1	0	1	8
Elmas 2025 (14)	1	1	1	1	1	1	1	1	1	9
Sahin 2026 (18)	1	1	1	1	1	1	1	1	1	9
Wu 2026 (19)	0	1	1	1	1	1	1	1	1	8
Liu 2026 (16)	1	1	1	1	1	1	1	0	1	8
Zhang 2026a (20)	0	1	1	1	1	1	1	1	1	8
Zhang 2026b (21)	1	1	1	1	1	1	1	0	1	8

### Results of the meta-analysis

**Figure 2:**
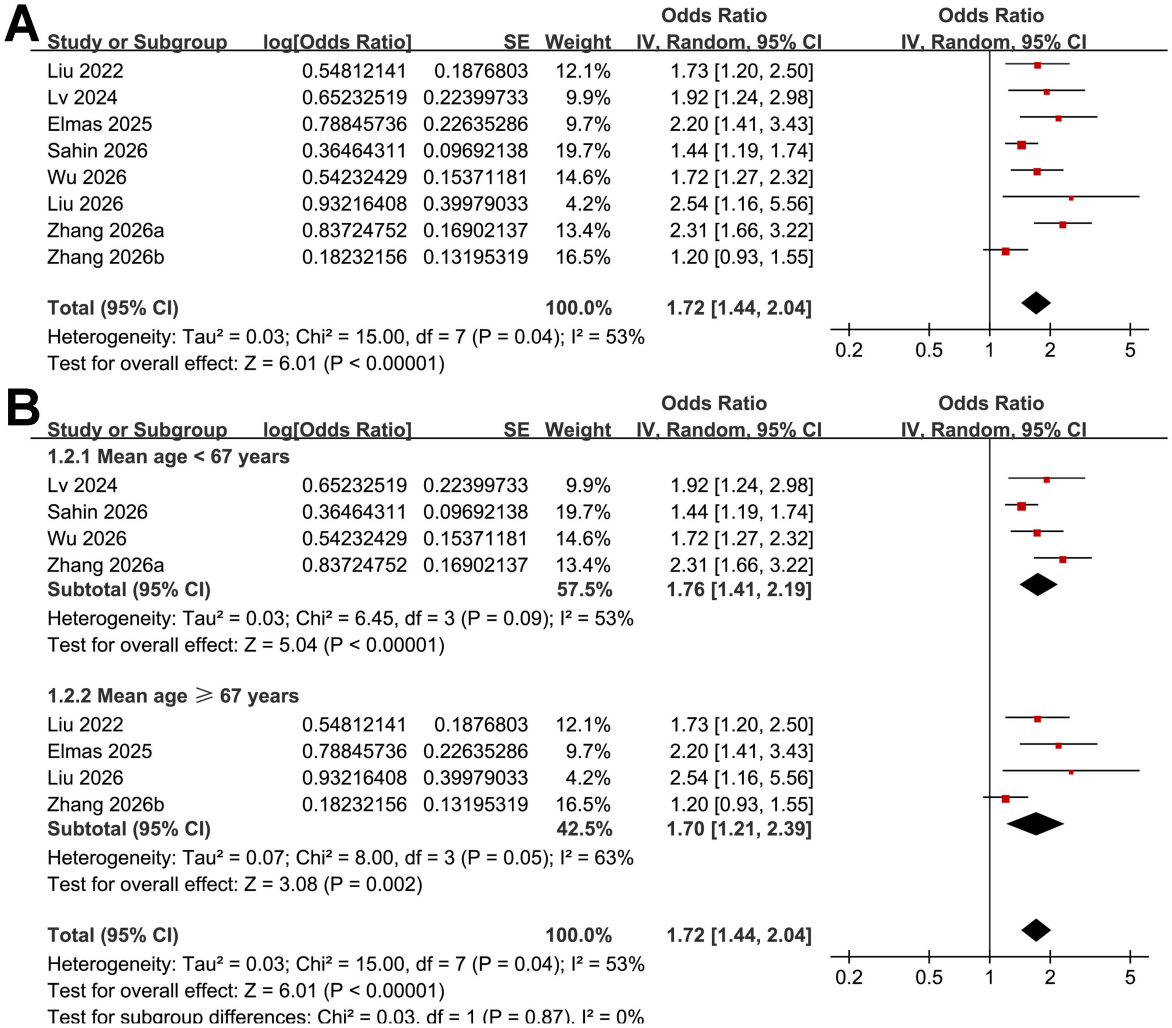
Forest plots showing the meta-analysis of the association between TyG index and short-term mortality of adult patients with sepsis: **(A)** forest plots for the overall meta-analysis; and **(B)** forest plots for the subgroup analysis according to the mean age of the patients.

The pooled analysis of the eight included studies (14–21) demonstrated that a higher TyG index at admission was associated with an increased risk of short-term mortality in adult patients with sepsis (OR: 1.72, 95% CI: 1.44 to 2.04, *p* < 0.001; **Figure 2A**) with moderate heterogeneity (Cochrane Q test *p* = 0.04; I² = 53%). Leave-one-out sensitivity analyses yielded consistent results, with pooled ORs ranging from 1.61 to 1.80 (all *p* < 0.05), indicating the robustness of the overall estimate.

### Results of the subgroup analysis

**Figure 3:**
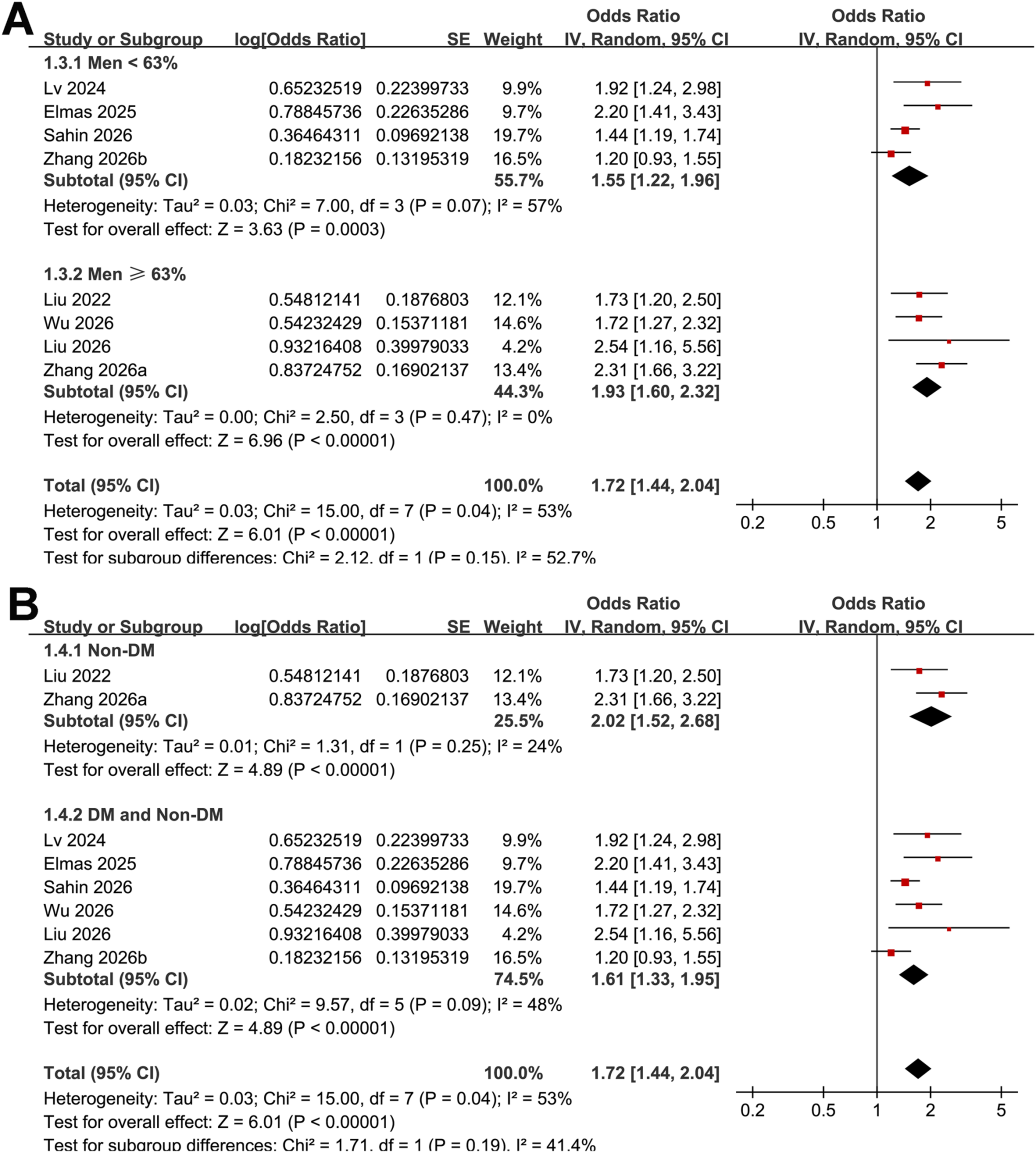
Forest plots showing the subgroup analysis of the association between TyG index and short-term mortality of adult patients with sepsis: **(A)** forest plots for the subgroup analysis according to the proportion of men; and **(B)** forest plots for the subgroup analysis according to the diabetic status of the patients.

**Figure 4:**
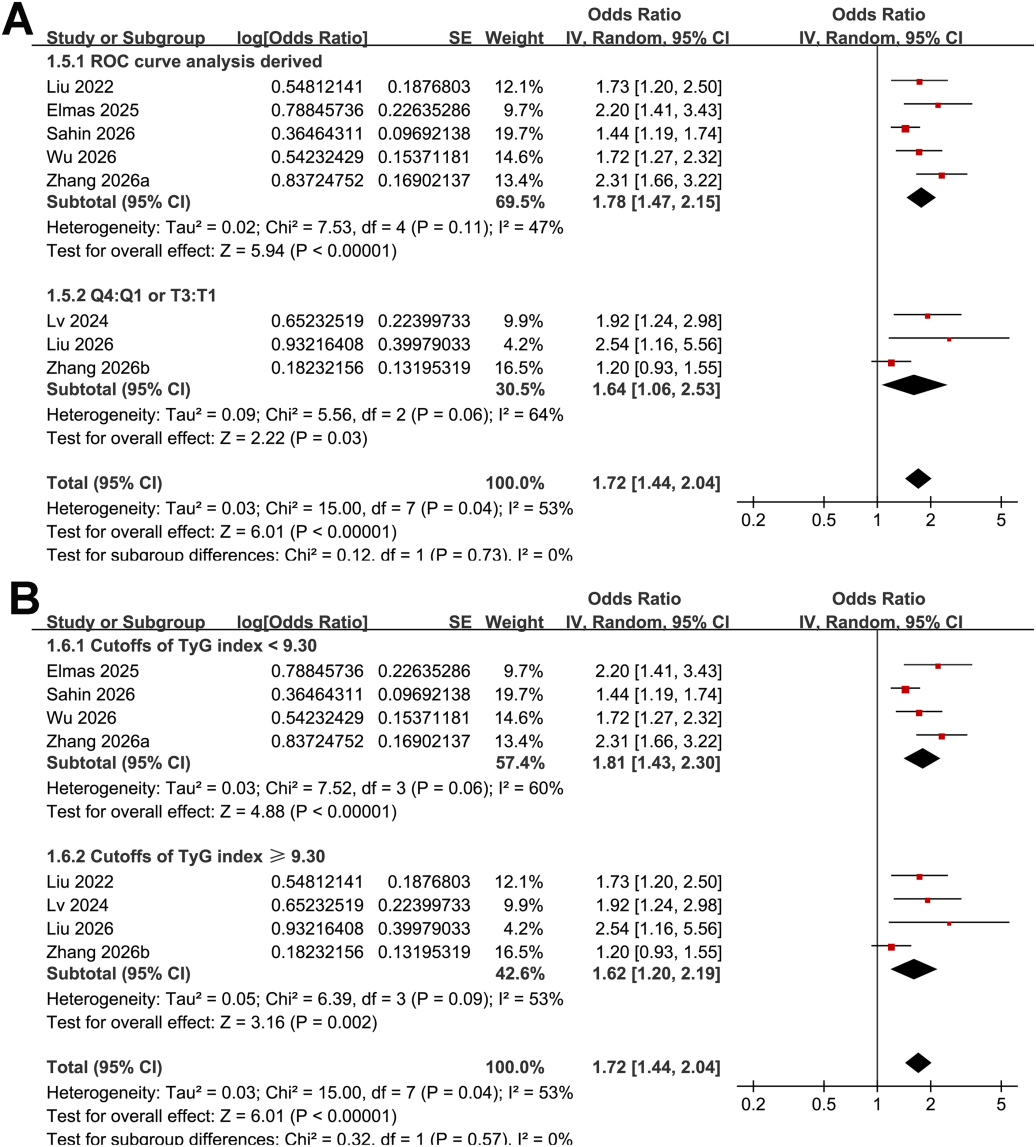
Forest plots showing the subgroup analysis of the association between TyG index and short-term mortality of adult patients with sepsis: **(A)** forest plots for the subgroup analysis according to the methods for determining the cutoffs of TyG index; and **(B)** forest plots for the subgroup analysis according to the cutoff values of TyG index.

**Figure 5:**
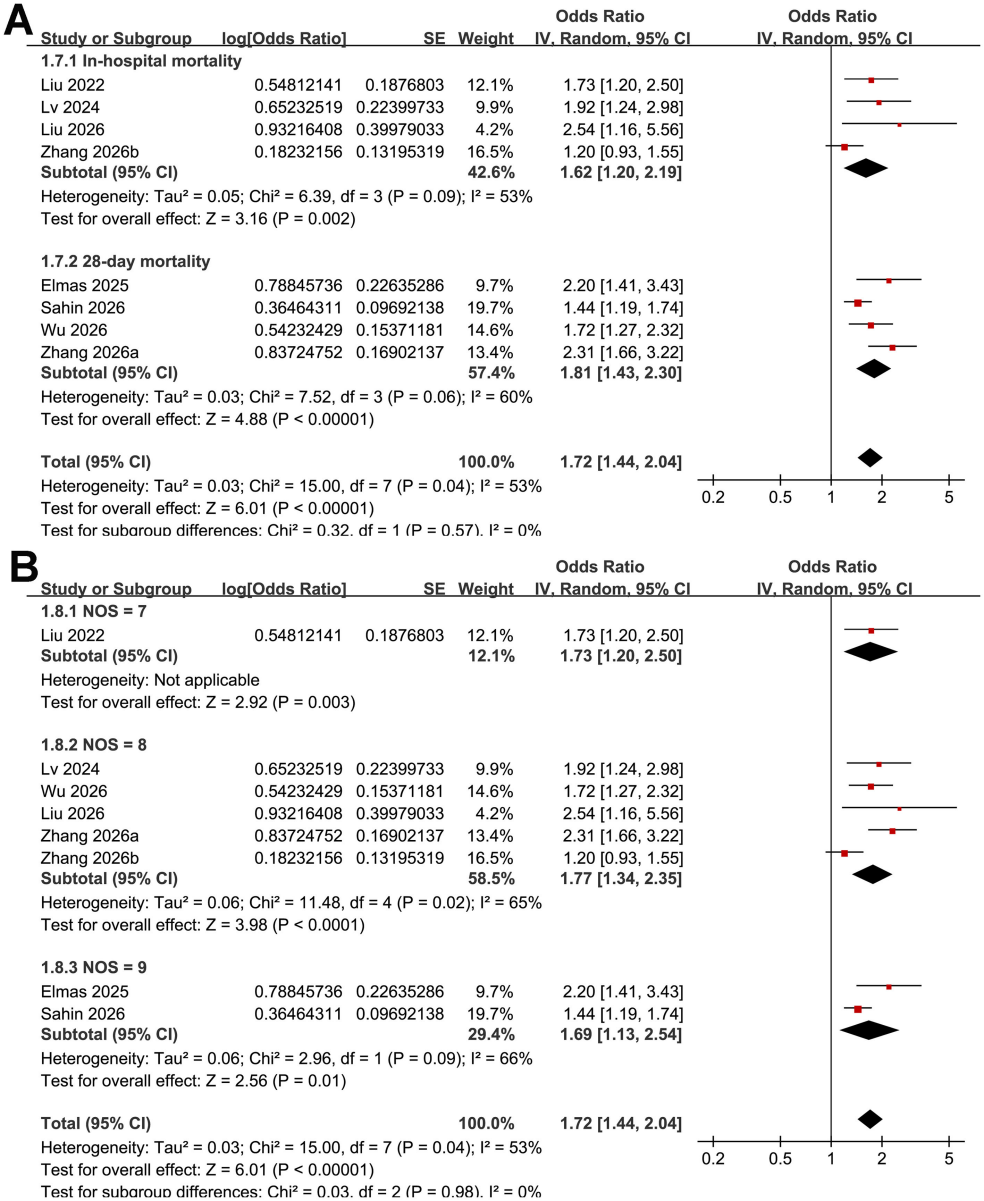
Forest plots showing the subgroup analysis of the association between TyG index and short-term mortality of adult patients with sepsis: **(A)** forest plots for the subgroup analysis according to the outcomes reported; and **(B)** forest plots for the subgroup analysis according to the study quality scores.

Subgroup analyses suggested a similar association between TyG index and short-term mortality of patients with sepsis in studies with mean patient ages < 67 and ≥ 67 years (OR: 1.76 vs. 1.70, *p* for subgroup difference = 0.87; **Figure 2B**), in populations with a proportion of men < 63% and ≥ 63% (OR: 1.55 vs. 1.93, *p* for subgroup difference = 0.15; **Figure 3A**), and between studies including non-diabetic only and those including both diabetic and non-diabetic patients (OR: 2.02 vs. 1.61, *p* for subgroup difference = 0.19; **Figure 3B**). Moreover, the results were also consistent between studies with cutoffs of TyG index determined by ROC curve analysis or quartiles/tertiles (OR: 1.78 vs. 1.64, *p* for subgroup difference = 0.73; **Figure 4A**), and between studies with the cutoff values of TyG index < 9.30 and ≥ 9.30 (OR: 1.81 vs. 1.62, *p* for subgroup difference = 0.57; **Figure 4B**). In addition, the results were similar between studies reporting in-hospital and 28-day mortality (OR: 1.62 vs. 1.81, *p* for subgroup difference = 0.57; **Figure 5A**), and in studies with the NOS scores of 7, 8, and 9 (OR: 1.73, 1.77, and 1.69, *p* for subgroup difference = 0.98; **Figure 5B**).

### Results of the meta-regression analysis

Univariate meta-regression analyses were performed to evaluate whether study-level characteristics were associated with variations in the effect size linking TyG index and short-term mortality in adult patients with sepsis, as summarized in **Table 3**. The results showed that none of the evaluated study characteristics significantly affected the association, including mean age, proportion of men, proportion of patients with diabetes, cutoff value of TyG index, or study quality scores (*p* all > 0.05; **Table 3**).

**Table 3:** Results of univariate meta-regression analysis.

Variables	Regression coefficient (β)	95% CI	P values	Adjusted R2
Mean age (years)	-0.0003	-0.0259 to 0.0254	0.980	0%
Men (%)	0.0021	-0.0233 to 0.0275	0.850	0%
DM (%)	-0.0016	-0.0131 to 0.0098	0.740	0%
Cutoff of TyG index	-0.1900	-0.9300 to 0.5600	0.560	0%
NOS score	-0.0240	-0.4130 to 0.3660	0.890	0%

### Publication bias

**Figure 6:**
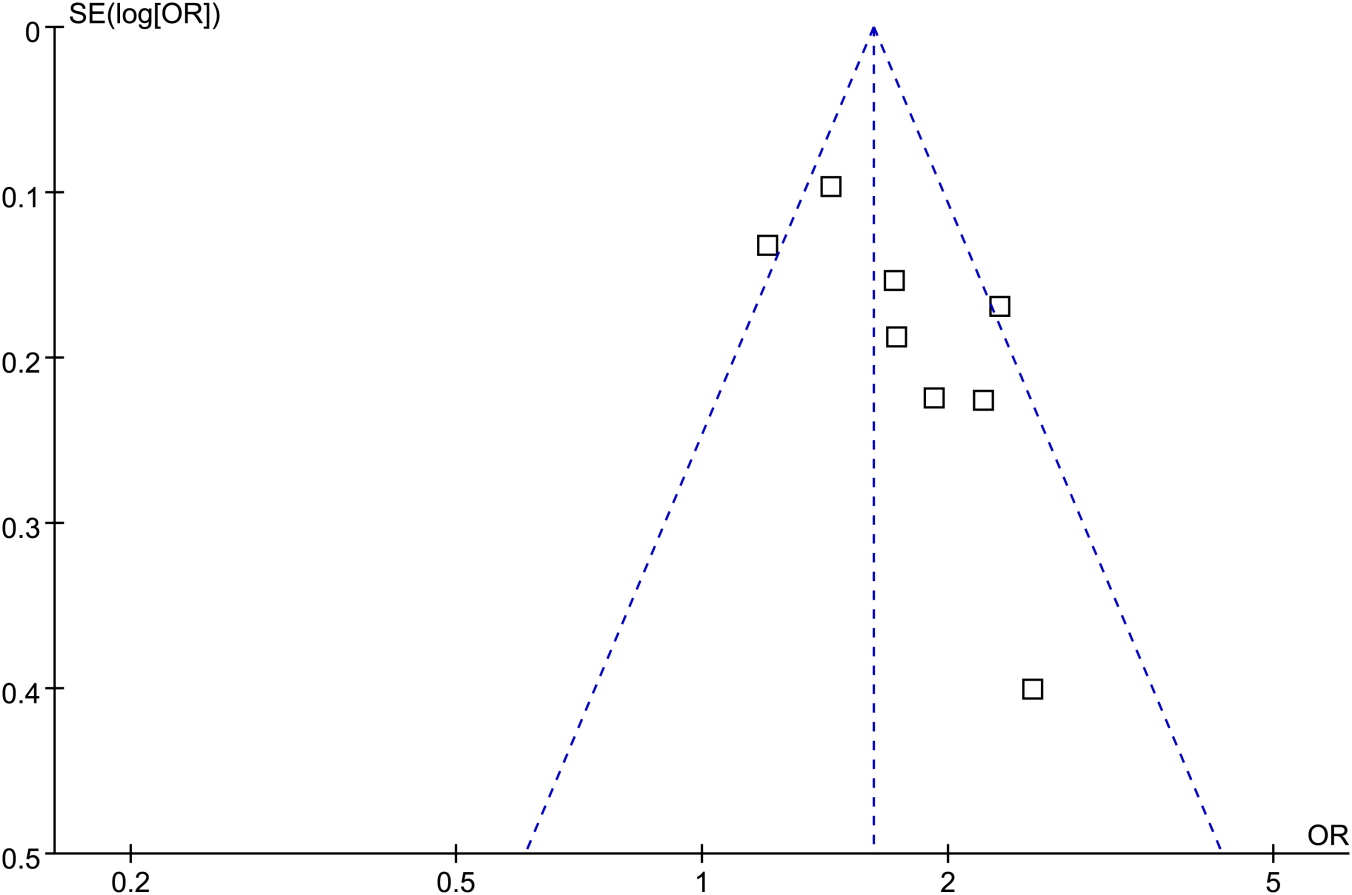
Funnel plots evaluating the publication bias for the meta-analysis of the association between TyG index and short-term mortality of adult patients with sepsis.

As illustrated in **Figure 6**, the funnel plots assessing the association between TyG index and short-term mortality of adult patients with sepsis appeared generally symmetrical. Egger’s regression test did not detect significant funnel plot asymmetry (*p* = 0.48). However, these findings should be interpreted cautiously because only eight studies were included. Statistical tests for publication bias have limited power when the number of studies is small, and therefore the possibility of publication bias cannot be reliably excluded despite the non-significant result.

## Discussion

This meta-analysis provides a comprehensive synthesis of current evidence regarding the prognostic value of the TyG index in adult patients with sepsis. By integrating data from multiple cohort studies, the findings suggest that a higher TyG index is associated with an increased risk of short-term mortality. Importantly, this association was robust across a range of sensitivity and subgroup analyses, and was not significantly modified by key study-level characteristics. These observations support the potential role of the TyG index as a clinically relevant biomarker reflecting adverse metabolic and inflammatory states in sepsis.

The biological plausibility of this association may be explained by the components of the TyG index, namely fasting triglycerides and glucose, both of which are closely linked to insulin resistance and metabolic dysfunction (10). In sepsis, profound alterations in glucose metabolism occur as part of the stress response, including increased gluconeogenesis, insulin resistance, and impaired glucose utilization (27, 28). Hyperglycemia has been associated with immune dysregulation, impaired neutrophil function, and increased susceptibility to infection, thereby potentially worsening outcomes (29, 30). Similarly, elevated triglyceride levels may reflect altered lipid metabolism and contribute to inflammatory responses (31). Lipids play a complex role in host defense, including the neutralization of bacterial toxins (32). However, dysregulated lipid metabolism may promote endothelial dysfunction, oxidative stress, and pro-inflammatory signaling (33). Therefore, a higher TyG index may represent an integrated marker of metabolic derangement, systemic inflammation, and immune dysfunction, all of which are key drivers of organ failure and mortality in sepsis. Recent evidence suggests that sepsis is characterized by profound immunometabolic dysregulation, in which inflammatory activation and neuroendocrine stress responses promote insulin resistance and altered energy metabolism (34, 35). These metabolic disturbances may further impair innate and adaptive immune function, exacerbate endothelial injury, and contribute to multiple organ dysfunction (34, 35). Accordingly, an elevated TyG index may identify patients with more severe metabolic and inflammatory derangements who are at increased risk of adverse outcomes.

The consistency of the observed association across subgroup analyses further strengthens the credibility of the findings. The lack of significant differences across age groups, sex distribution, diabetic status, and study quality suggests that the prognostic value of the TyG index is relatively stable across diverse patient populations. Notably, the association was observed both in studies including only non-diabetic patients and in those including mixed populations, indicating that the TyG index may capture metabolic risk beyond the presence of overt diabetes. Similarly, consistent results across different methods of defining TyG cutoffs, including ROC-derived thresholds and quantile-based comparisons, suggest that the observed association is not dependent on a specific categorization strategy. However, the cutoff values used to define a high TyG index varied considerably across studies, ranging from 8.54 to 9.55, which may limit direct clinical implementation. Potential sources of this variability include differences in patient demographics, prevalence of diabetes and other metabolic disorders, severity of sepsis, timing of blood sampling, laboratory measurement conditions, and statistical approaches used to determine thresholds. Although our subgroup analyses showed similar associations across studies with lower and higher cutoff values and across different cutoff determination methods, the absence of a standardized threshold remains an important challenge for clinical application. Future prospective multicenter studies should aim to establish and externally validate unified TyG index cutoffs specifically for patients with sepsis using standardized measurement protocols and outcome-based approaches. Such efforts may improve the utility of the TyG index for bedside risk stratification and facilitate its integration into routine prognostic assessment. The meta-regression analyses did not identify significant modifiers of the observed association. However, these findings should be interpreted cautiously because the statistical power of meta-regression is limited when only a small number of studies are available.

Several strengths of this meta-analysis should be highlighted. First, this study incorporated an up-to-date and comprehensive literature search across multiple international and Chinese databases, enhancing the completeness of evidence retrieval. Second, all included studies adopted a cohort design with longitudinal follow-up, which is appropriate for evaluating prognostic associations. Third, the analysis was based on multivariable-adjusted estimates, thereby reducing the impact of confounding factors and improving the reliability of the pooled results. Additionally, the use of multiple sensitivity, subgroup, and meta-regression analyses provides a thorough evaluation of the robustness and potential sources of heterogeneity.

Nevertheless, several limitations should be considered when interpreting the findings. All included studies were single-center retrospective cohort studies, which may introduce selection and information biases and limit the ability to establish causal relationships. The retrospective design also raises the possibility of residual confounding, as not all relevant clinical variables may have been measured or adequately adjusted for in the original studies (36). Furthermore, the absence of prospective and multicenter data may restrict the external validity and generalizability of the findings across different patient populations and healthcare settings. Therefore, the current results should be interpreted cautiously until confirmed by large prospective multicenter studies. In addition, some included studies had relatively small sample sizes and limited numbers of outcome events despite adjustment for multiple covariates. Under such circumstances, multivariable models may be susceptible to overfitting, which could affect the precision of estimated effect sizes and potentially influence study weights in meta-analysis. Although our leave-one-out sensitivity analyses demonstrated that the pooled association remained stable after sequential exclusion of individual studies, suggesting that no single study materially influenced the overall findings, the possibility of bias related to over-fitted primary models cannot be completely excluded. Therefore, results from small studies with heavily adjusted models should be interpreted with appropriate caution. Moreover, although moderate heterogeneity was observed, our ability to explore potential sources of heterogeneity was limited by the availability of study-level data. The timing of TyG index assessment was relatively consistent across studies, being performed either within 24 hours of admission or at the first fasting blood sampling after admission. However, detailed information regarding laboratory testing protocols for triglyceride and glucose measurements was generally unavailable. In addition, while most studies adjusted for disease severity using established indicators such as SOFA or APACHE II scores, stratified outcome data according to sepsis severity were not reported. Consequently, further analyses of these potentially important sources of heterogeneity were not feasible. Future studies should adopt more standardized reporting of TyG measurement procedures and severity-specific results to facilitate more comprehensive evidence synthesis. Additionally, although 14,772 patients were included overall, only eight independent studies were available for quantitative synthesis. Notably, many recently published studies in this field were excluded because they were based on overlapping populations derived from the MIMIC-IV database, and only the largest dataset was retained to avoid duplication of participants. While this approach enhanced methodological rigor, it reduced the number of independent studies available for analysis. Consequently, the statistical power of subgroup analyses, meta-regression, and publication bias assessment was limited, which may have constrained our ability to identify true sources of heterogeneity and fully evaluate the influence of study-level characteristics. Although no significant funnel plot asymmetry was detected, the possibility of publication bias cannot be reliably excluded. Therefore, the current findings should be interpreted cautiously, and additional prospective studies and independent external cohorts are needed to further validate the observed associations. Moreover, the geographic distribution of the included studies was uneven. Although studies from China, Turkey, and the United States were included, most of the available evidence originated from Asian populations, with limited representation from other regions. Differences in ethnicity, healthcare systems, clinical management strategies, and population characteristics may influence the generalizability of the findings. Therefore, further validation in diverse geographic and ethnic populations is warranted. Finally, differences in laboratory measurement conditions may also have contributed to variability across studies. Sepsis is characterized by profound metabolic disturbances, including stress-induced hyperglycemia, increased insulin resistance, and altered lipid metabolism, which may affect the TyG index independently of pre-existing metabolic status (37). Moreover, early therapeutic interventions, such as aggressive fluid resuscitation, insulin administration, glucose-containing infusions, and nutritional support, may dynamically influence serum glucose and triglyceride concentrations (38). Future prospective studies using standardized protocols for TyG assessment and carefully accounting for early therapeutic interventions are warranted to better define the prognostic significance of the TyG index in patients with sepsis.

From a clinical perspective, the TyG index has several attractive features as a prognostic marker in sepsis. It is simple, inexpensive, and routinely available from standard laboratory tests, making it feasible for early risk stratification in a wide range of healthcare settings (39). The findings of this meta-analysis suggest that incorporating of the TyG index into clinical assessment may facilitate early identification of high-risk patients of short-term mortality, potentially facilitating more timely and targeted interventions. However, given the observational nature of the available evidence and the aforementioned limitations, these results should be interpreted with caution. The TyG index should be considered as a complementary tool rather than a replacement for established clinical scoring systems. Future research is warranted to further clarify the role of the TyG index in sepsis. Prospective, multicenter studies with standardized measurement protocols are needed to validate the prognostic value of the TyG index and to determine optimal cutoff values. Studies incorporating individual participant data may allow for more precise adjustment for confounding factors and exploration of interactions with patient characteristics. In addition, mechanistic studies are needed to elucidate the biological pathways linking metabolic dysfunction, as reflected by the TyG index, to adverse outcomes in sepsis. Ultimately, interventional studies may help determine whether targeting metabolic abnormalities can improve outcomes in this population.

## Conclusions

In conclusion, this meta-analysis suggests that a higher TyG index is associated with an increased risk of short-term mortality in adult patients with sepsis. These findings highlight the potential utility of the TyG index as a simple and accessible prognostic marker, while underscoring the need for further high-quality research to confirm its clinical applicability.

## Data Availability

The original contributions presented in the study are included in the article/**Supplementary Material**. Further inquiries can be directed to the corresponding author.

## Glossary

NR: not reported
RC: retrospective cohort
DM: diabetes mellitus
TyG: triglyceride-glucose
ROC: receiver operating characteristic
Q1: first quartile
Q4: fourth quartile
T1: first tertile
T3: third tertile
HR: heart rate
MAP: mean arterial pressure
PCT: procalcitonin
CRP: C-reactive protein
LAC: lactate
IL-6: interleukin-6
MV: mechanical ventilation
SOFA: Sequential Organ Failure Assessment
APACHE II: Acute Physiology and Chronic Health Evaluation II
BMI: body mass index
ALB: albumin
HbA1c: glycated hemoglobin
HDL-C: high-density lipoprotein cholesterol
CAD: coronary artery disease
HBG: hemoglobin
PLT: platelet count
WBC: white blood cell count
TBIL: total bilirubin
HCT: hematocrit
TyG: triglyceride-glucose
CI: confidence interval
DM: diabetes mellitus
NOS: Newcastle-Ottawa Scale
