# Supplementary material for: Triglyceride-glucose index and short-term mortality of adult patients with sepsis: a meta-analysis
Source: Front Endocrinol (Lausanne). 2026 Jul 30;17:1875452. doi: 10.3389/fendo.2026.1875452 (PMC13467863; doi:10.3389/fendo.2026.1875452)
Supplement: Supplementary file 1 [file DataSheet1.docx]

**Detailed search strategy for each database**

**PubMed**

("triglyceride-glucose index"[Title/Abstract] OR "triglyceride glucose index"[Title/Abstract] OR "triglyceride and glucose index"[Title/Abstract] OR "triacylglycerol glucose index"[Title/Abstract] OR "TyG index"[Title/Abstract] OR TyG[Title/Abstract]) AND ("Sepsis"[Mesh] OR "Septicemia"[Mesh] OR sepsis[Title/Abstract] OR septic[Title/Abstract] OR septicemia[Title/Abstract] OR septicaemia[Title/Abstract])

**Embase**

('triglyceride glucose index':ti,ab,kw OR 'triglyceride-glucose index':ti,ab,kw OR 'triglyceride and glucose index':ti,ab,kw OR 'triacylglycerol glucose index':ti,ab,kw OR 'tyg index':ti,ab,kw OR tyg:ti,ab,kw) AND ('sepsis'/exp OR 'septicemia'/exp OR sepsis:ti,ab,kw OR septic:ti,ab,kw OR septicemia:ti,ab,kw OR septicaemia:ti,ab,kw)

**Web of Science**

TS=("triglyceride-glucose index" OR "triglyceride glucose index" OR "triglyceride and glucose index" OR "triacylglycerol glucose index" OR "TyG index" OR TyG) AND TS=(sepsis OR septic OR septicemia OR septicaemia)

**Wanfang**

主题 = (("TyG指数" OR "甘油三酯葡萄糖指数" OR "甘油三酯-葡萄糖指数" OR "甘油三酯/葡萄糖指数" OR "三酰甘油葡萄糖指数" OR "三酰甘油-葡萄糖指数") AND ("脓毒症" OR "败血症" OR "感染性休克"))

**CNKI**

SU = (("TyG指数" OR "甘油三酯葡萄糖指数" OR "甘油三酯-葡萄糖指数" OR "甘油三酯/葡萄糖指数" OR "三酰甘油葡萄糖指数" OR "三酰甘油-葡萄糖指数") AND ("脓毒症" OR "败血症" OR "感染性休克"))
