# Supplementary material for: Triglyceride-glucose index and short-term mortality of adult patients with sepsis: a meta-analysis
Source: Front Endocrinol (Lausanne). 2026 Jul 30;17:1875452. doi: 10.3389/fendo.2026.1875452 (PMC13467863; doi:10.3389/fendo.2026.1875452)
Supplement: Supplementary file 2 [file DataSheet2.docx]

**Studies excluded in full-text review**

**Including pediatric patients (n = 1)**

- Triglyceride-Glucose Index and 30-day mortality in pediatric sepsis: a retrospective cohort study based on PIC database. Front Pediatr. 2026 Feb 27:14:1704208. doi: 10.3389/fped.2026.1704208.

**Not in patients with sepsis (n = 1)**

- Associations of the triglyceride-glucose index with short-term mortality in patients with cardiogenic shock: a cohort study. Lipids Health Dis. 2025 Apr 4;24(1):130. doi: 10.1186/s12944-025-02548-z.

**TyG index not measured (n = 4)**

- Associations between triglyceride-glucose body mass index and all-cause mortality in ICU patients with sepsis and acute heart failure. BMC Cardiovasc Disord. 2025 May 9;25(1):359. doi: 10.1186/s12872-025-04804-7.
- U-shaped association between triglyceride/HDL ratio and all-cause mortality in obese sepsis patients: A retrospective study based on eICU database. Med Intensiva (Engl Ed). 2026 Jun;50(6):502308. doi: 10.1016/j.medine.2025.502308.
- Triglyceride-to-high-density lipoprotein ratio as a predictor of 28-day mortality risk in patients with Sepsis: A retrospective cohort study. Respir Med. 2025 Nov:248:108419. doi: 10.1016/j.rmed.2025.108419.
- Association between triglyceride glucose-body mass index and all-cause mortality in patients with sepsis: Evidence from a retrospective cohort study. J Int Med Res. 2026 Feb;54(2):3000605261420914. doi: 10.1177/03000605261420914.

**Short-term mortality outcome not reported (n = 2)**

- Comparison of the predictive value of different non-insulin-based insulin resistance indices for acute kidney injury in patients with sepsis: a retrospective study. Front Endocrinol (Lausanne). 2025 Nov 18:16:1637119. doi: 10.3389/fendo.2025.1637119.
- Association of triglyceride glucose index with sepsis risk after major abdominal surgery: A retrospective cohort study. Pak J Med Sci. 2025 Jun;41(6):1734-1742. doi: 10.12669/pjms.41.6.12187.

**Studies with overlapping patients (n = 11)**

- Association between triglyceride-glucose index and in-hospital mortality in critically ill patients with sepsis: analysis of the MIMIC-IV database. Cardiovasc Diabetol. 2023 Nov 8;22(1):307. doi: 10.1186/s12933-023-02041-w.
- The triglyceride-glucose index dynamic trajectory reveals the association between the clinical subphenotype of insulin resistance and mortality in patients with sepsis. BMC Infect Dis. 2024 Oct 1;24(1):1083. doi: 10.1186/s12879-024-10005-y.
- Impact of the triglyceride-glucose index on 28-day mortality in non-diabetic critically Ill patients with sepsis: a retrospective cohort analysis. BMC Infect Dis. 2024 Aug 5;24(1):785. doi: 10.1186/s12879-024-09711-4.
- Triglyceride-glucose index and clinical outcomes in sepsis: A retrospective cohort study of MIMIC-IV. J Cell Mol Med. 2024 Aug;28(16):e70007. doi: 10.1111/jcmm.70007.
- A retrospective study utilized MIMIC-IV database to explore the potential association between triglyceride-glucose index and mortality in critically ill patients with sepsis. Sci Rep. 2024 Oct 15;14(1):24081. doi: 10.1038/s41598-024-75050-8.
- The association between the triglyceride‒glucose index and short-term mortality in ICU patients with sepsis-associated acute kidney injury. BMC Infect Dis. 2025 Feb 24;25(1):257. doi: 10.1186/s12879-025-10649-4.
- Association between the TyG index and the risk of in-hospital mortality from early sepsis-related acute kidney injury in critically ill patients: a secondary analysis of MIMIC-IV 2008-2022. BMJ Open. 2025 May 7;15(5):e099529. doi: 10.1136/bmjopen-2025-099529.
- Joint association of the triglyceride-glucose index and stress hyperglycemia ratio with incidence and mortality risks of new-onset atrial fibrillation during sepsis: a retrospective cohort study. Cardiovasc Diabetol. 2025 Apr 2;24(1):149. doi: 10.1186/s12933-025-02709-5.
- Triglyceride-glucose index: a novel prognostic marker for sepsis-associated encephalopathy severity and outcomes. Front Neurol. 2025 Apr 2:16:1468419. doi: 10.3389/fneur.2025.1468419.
- Association of TyG index with mortality at 28 days in sepsis patients in intensive care from MIMIC IV database. Sci Rep. 2025 Jan 17;15(1):2344. doi: 10.1038/s41598-025-86746-w.
- The role of triglyceride-glucose (TyG) index in predicting mortality risk in critically ill patients with sepsis: a machine learning model in a cohort study. BMC Med Inform Decis Mak. 2026 Apr 20. doi: 10.1186/s12911-026-03502-4. Online ahead of print.
